# The clinical value of PDCA cycle led by clinical pharmacists in optimizing the use of antimicrobial drugs in pediatric departments of primary hospitals

**DOI:** 10.3389/fmed.2026.1917853

**Published:** 2026-08-18

**Authors:** Yunxi Zhao, Yuhan Tu, Lingzhi Zhao, Chuanfeng Shao, Peilei Chen

**Affiliations:** 1 Department of Pharmacy, Yueqing People's Hospital, Wenzhou, China; 2 Department of Pharmacy, Yueqing Third People's Hospital, Wenzhou, China; 3 Pediatrics, Yueqing Third People's Hospital, Wenzhou, China; 4 School of Pharmacy, Wenzhou Medical University, Wenzhou, China

**Keywords:** antimicrobial drugs, clinical pharmacy, PDCA cycle, pediatrics, primary hospitals

## Abstract

**Background:**

Pediatric departments in primary hospitals face challenges such as insufficient diagnostic capabilities, weak management systems, and a lack of guidance on medication use. These factors have led to persistently high rates of antimicrobial use and misuse, which seriously jeopardize the safety of pediatric care in primary hospitals. Clinical pharmacists play a key role in ensuring the appropriate use of medications. However, in primary hospitals, their role is often marginalized or entirely absent.

**Objective:**

This study aims to evaluate the effectiveness of a multidisciplinary antimicrobial stewardship intervention led by clinical pharmacists in promoting the appropriate use of antimicrobials among pediatric outpatients and inpatients at a primary care hospital.

**Method:**

This study established a multidisciplinary team led by clinical pharmacists and conducted a quality improvement study in the pediatric department of a primary care hospital. ChatGPT was introduced as a brainstorming tool during the root cause analysis process. Interventions included daily embedded ward rounds, electronic prescription preauthorization, and structured training. After two Plan-Do-Check-Act (PDCA) cycles, changes in primary and secondary outcome measures were compared between the control group and the two intervention groups.

**Results:**

After two PDCA cycles, the outpatient antimicrobial usage rate decreased from 38.81 to 27.14%, the intravenous infusion rate decreased from 28.29 to 20.04%, and the per-patient medication cost decreased from ¥68.50 to ¥49.73 (*p* < 0.0001). For inpatients, the antibiotic usage rate decreased from 90.92 to 78.36%, the antibiotic intensity decreased from 82.24 to 49.94, and the perpatient medication cost decreased from 281.94 Yuan to 130.90 Yuan (*p* < 0.0001). The rate of inappropriate prescribing among outpatients decreased from 34.75 to 12.08%, and among inpatients, it decreased from 40.30 to 13.42% (*p* < 0.0001). No statistically significant change was observed in the average length of hospital stay (*p* > 0.05). A standardized prescribing workflow was established.

**Conclusion:**

Antimicrobial utilization decreased following implementation of the intervention. Tailored to the practical realities and characteristics of primary healthcare settings, it demonstrates both feasibility and replicability. It provides evidence-based guidance and a management framework for advancing rational drug use in children at the primary care level.

## 1 Introduction

The misuse and overuse of antimicrobial drugs have become a severe and escalating threat to global public health, accelerating the development of antimicrobial resistance (AMR) and significantly undermining humanity’s ability to effectively treat infectious diseases (1). According to The Lancet’s projections, by 2050 1.91 million deaths will be attributable to AMR each year, while an additional 8.22 million deaths will be associated with AMR (31). The standardized management of antimicrobial agents has become one of the core issues in international healthcare quality improvement (2, 3). Pediatrics, as a key area for antimicrobial use, presents unique and complex challenges to rational antimicrobial stewardship due to factors such as significant differences in disease spectrum, pharmacokinetics, and pharmacodynamics compared to adults, as well as greater diagnostic and therapeutic complexity (4).

The Guidelines for the Rational Use of Medications for Acute Respiratory Infections in Children indicate that among children hospitalized for respiratory infections in China, 83.0% were prescribed antimicrobial agents upon admission. However, the rate of rational prescribing of antimicrobial agents in primary hospital is only 14.3% (5). In accordance with the requirements of the Administrative Measures for the Clinical Use of Antimicrobials issued by the National Health Commission of China, Level II general hospitals should maintain the outpatient antimicrobial usage rate below 20%, the inpatient antimicrobial usage rate below 60%, and the antimicrobial usage intensity below 40 DDD (6, 7). Although pediatric departments already have high levels of antimicrobial use due to the prevalence of infectious diseases among children, the actual indicators in pediatric departments at primary hospitals generally far exceed reasonable benchmarks. The core driver of this is not the case mix, but rather inappropriate prescribing practices. This widespread practice of defensive or empirical prescribing not only deprives most children with viral infections of effective treatment but may also affect their growth and development. At the same time, this practice exacerbates bacterial resistance, increases the incidence of adverse drug reactions, and leads to a waste of medical resources (8, 9).

Despite the publication of numerous clinical practice guidelines and management standards for pediatric antimicrobial use both domestically and internationally, providing a theoretical basis for clinical decision-making (10). However, in the actual clinical practice of primary hospitals, factors such as uneven distribution of medical resources, insufficient staffing of pediatric specialists, weak management capabilities for antimicrobial use, inadequate guidance systems for rational medication, and the absence of sustainable quality improvement mechanisms have constrained efforts (11). The management outcomes of rational antimicrobial use in pediatrics remain difficult to consolidate, with persistent challenges in controlling antimicrobial usage rates, intensity, and costs (10). Furthermore, most antimicrobial stewardship programs rely on periodic administrative inspections rather than embedded, continuous quality improvement mechanisms (12, 13). Therefore, exploring a practical and sustainable model for pediatric antimicrobial stewardship that takes into account the characteristics of primary hospitals, and optimizing the use of antimicrobials in children at primary facilities, has become an urgent task in the fields of clinical pharmacy and pediatric care at this stage.

The Plan-Do-Check-Act (PDCA) Cycle, as a classic quality management tool, provides a scientific framework for continuous improvement in healthcare quality through its four-stage spiral progression: plan, do, check, and act. Its evidence-based and continuous nature makes it highly suitable for the ongoing optimization of antimicrobial stewardship (14). Currently, the PDCA cycle has been validated as effective in multiple medical settings, including self-management for patients with gestational diabetes (15), infection control at craniotomy sites in neurosurgery (16), blood glucose management for critically ill patients (17), and nutritional management for nasopharyngeal carcinoma patients (18). However, these studies have primarily focused on well-resourced tertiary hospitals, failing to adequately incorporate the characteristics of primary hospitals in terms of resources, personnel, and diagnostic and treatment conditions (19). The lack of systematic exploration into antimicrobial use in primary pediatric care has resulted in existing management models being poorly suited to primary hospitals. This prevents the core issue of inappropriate antimicrobial use in primary pediatric settings from being effectively addressed, leaving the current situation of antimicrobial abuse in children at primary hospitals fundamentally unresolved (20).

The root cause analysis phase of the traditional PDCA cycle heavily relies on brainstorming among project team members. This approach is susceptible to limitations imposed by individual experience and cognitive perspectives, often resulting in incomplete and imprecise analyses. Consequently, this compromises the precision and effectiveness of subsequent corrective actions (21). The rapid advancement of artificial intelligence technology in recent years has opened new avenues to address this limitation. Large-scale language models, exemplified by ChatGPT 4.0, leverage their capabilities in integrating multi-source knowledge and conducting multidimensional analysis to broaden the scope of root cause analysis discussions. This enhances the comprehensiveness and innovation of brainstorming sessions, injecting fresh vitality into traditional quality management practices (22).

As professionals well-versed in drug characteristics and rational drug use practices, clinical pharmacists play a crucial role in ensuring patient medication safety and improving the effectiveness of drug therapy (23). However, county-level primary hospitals in China generally lack a sustainable antimicrobial stewardship model led by pharmacists and tailored to the mixed outpatient and inpatient pediatric care setting. Furthermore, due to limited professional capacity at the primary level, traditional quality improvement tools fall short in identifying root causes. To address this gap, this study aims to develop and validate an antimicrobial stewardship model in the pediatric department of a county-level Grade II Class A hospital in China. This model is led by clinical pharmacists, relies on artificial intelligence to assist in root cause analysis, and is implemented through sequential PDCA cycles. The primary objective of this study is to evaluate the effectiveness of this intervention model in improving antimicrobial usage rates among pediatric outpatients and inpatients, while the secondary objective is to assess improvements in antimicrobial-use-related secondary indicators, including antimicrobial usage intensity, per-capita antimicrobial expenditure, and the proportion of total healthcare costs attributed to antimicrobials. Through two rounds of PDCA cycles, this study established and standardized an antimicrobial prescribing process suitable for pediatric departments in primary hospitals, thereby significantly improving the rationality of medication use and reducing the unnecessary consumption of medical resources. In summary, this study aims to establish a scientific, refined, and sustainable pediatric antimicrobial management model for primary hospitals and provide a replicable management process to continuously enhance the rationality of medication use.

## 2 Materials and methods

### 2.1 Study design

This study was a single-center quality improvement project conducted in the real-world clinical setting of a county-level general hospital in a Chinese city that has been designated as a Grade II, Class A hospital. The hospital has a total of 660 beds, of which 46 are in the pediatric department. The department is staffed by 8 pediatricians and 11 nurses, and the average monthly number of pediatric inpatients is approximately 250. According to the Chinese Hospital Accreditation System, a Grade II, Class A hospital is a secondary-level medical institution with 100 to 500 beds that serves as a medical center for a county or district. The hospital provides multidisciplinary outpatient consultations and inpatient services. Its functions are equivalent to those of a district-level general hospital or county hospital in the international healthcare system.

The core framework of the project is the PDCA cycle. By employing a non-randomized, pre- and post-intervention comparative design, the effectiveness and sustainability of the intervention measures were assessed through comparing changes in key performance indicators before and after the PDCA cycle. The study followed the SQUIRE 2.0 guidelines.

Since this study focuses on quality improvement principles for the daily work of healthcare professionals, all pediatric outpatients and inpatients will be included in the analysis. Children receiving outpatient and inpatient treatment between January and July 2024 were assigned to the outpatient Control group (*n* = 37,145) and inpatient Control group (*n* = 1,531), respectively. From September 2024 to March 2025, pediatric outpatients and inpatients were assigned to the first-round PDCA cycle intervention group (Outpatient-1 group (*n* = 33,158), Inpatient-1 group (*n* = 1,301)). From May 2025 to November 2025, pediatric outpatients and inpatients were assigned to the Second-round PDCA cycle intervention group (Outpatient-2 group (*n* = 31,917), Inpatient-2 group (*n* = 1,266)). A one-month effect buffer period is established between the two cycles to prevent cross-interference between intervention measures.

### 2.2 Intervention measures

Establish a pediatric antimicrobial stewardship continuous quality improvement team. The team will be led by a clinical pharmacist and comprise members including the pediatric medical team leader, clinical physicians, infection control nurses, and pharmacy pharmacists. The team will develop standardized operational protocols, hold monthly progress meetings, and conduct quarterly phase reviews. The specific PDCA cycle implementation steps are as follows:

#### 2.2.1 Plan

The research team extracted data from the hospital information system (HIS) for the control group and conducted an analysis. The baseline indicators (January to July 2024) are as follows: The outpatient antimicrobial usage rate was 38.81%, the outpatient antimicrobial infusion rate was 28.29%, the average medication cost per outpatient visit was 68.50 yuan, the inpatient antimicrobial usage rate (AUR) was 90.92%, and the inpatient antimicrobial usage volume (AUD) was 82.24 DDD per 100 patient-days. A comparative analysis of these indicators against China’s national standards reveals that pediatric departments in hospitals face issues such as high rates of antimicrobial use, a significant proportion of drug costs in total healthcare spending, high intensity of antimicrobial use, and high rates of outpatient intravenous administration of antimicrobials. These issues clearly indicate that there are many instances of irrational antimicrobial use in pediatric departments at primary hospitals.

To broaden the scope of root cause analysis while reducing the risk of cognitive fixation associated with unstructured team brainstorming and minimizing the likelihood of overlooking valid causal dimensions, this study utilized ChatGPT-4.0 (OpenAI, accessed August 2024) as a brainstorming aid. This study designed a standardized initial prompt requesting the generation of a comprehensive, multidimensional list of potential factors influencing the inappropriate use of antimicrobial agents in children at primary hospitals. This list was clearly divided into four predefined domains: personnel, methods, environment, and management. The generated output was recorded verbatim and compiled directly into a preliminary list of factors without any manual modification (as shown in Supplementary material 1). Subsequently, the AI-generated list was consolidated and screened by a multidisciplinary team during a formal brainstorming session. The project team then designed a questionnaire and distributed it to six core team members, who rated the items using a “5–3-1” scoring method (5 = extremely important, 3 = relatively important, 1 = general factor) (Supplementary Tables S1, S2). All candidate factors were validated through actual clinical practice, and following consensus reached by the multidisciplinary clinical team, the root causes of inappropriate antimicrobial use in pediatrics were finally identified.

In summary, the methodology designed for this study involved first using AI to generate all possible scenarios, followed by team members taking the lead in validating these scenarios based on the actual conditions at the hospital, thereby ensuring that AI was used solely to augment, rather than replace clinical judgment and real-world circumstances. The exact prompts, interaction structure, and raw output generated by ChatGPT-4.0 are provided in the Supplementary Methods.

#### 2.2.2 DO

This phase represents a critical process for translating plans into concrete actions, with the core focus on systematically transforming clinical practices through the deep integration and specialized interventions of clinical pharmacists. The following five major measures will be implemented:

(1) Clinical pharmacists conduct daily embedded rounds and implement interventions

Clinical pharmacists are embedded as permanent members in pediatric daily morning handover meetings and ward rounds, with each round lasting no less than 1 h, focusing particularly on pediatric patients receiving antimicrobial therapy (24). During rounds, pharmacists conduct real-time reviews of medication indications. For cases lacking clear evidence of bacterial infection (e.g., absence of supporting blood counts, C-reactive protein levels, or pathogen testing), pharmacists recommend suspending antimicrobial drugs use to the attending physician and document this decision at the patient’s bedside. For patients who genuinely require medication, based on authoritative guidelines such as the “Diagnosis and Treatment Specifications for Community-Acquired Pneumonia in Children” and the “Guidelines for Rational Medication Use in Acute Respiratory Infections in Children” personalized recommendations are provided to physicians regarding drug selection, dosage conversion, and treatment duration. These recommendations adhere to the principles of “narrow rather than broad spectrum, single rather than combination therapy, and low rather than high dosage” ensuring the rationality of medication use (25). Clinical pharmacists document daily interventions in the Antimicrobial Stewardship Intervention Log, recording cases, intervention details, and physician adoption rates. Concurrently, they provide one-on-one medication education to parents, explaining antimicrobial usage protocols, adverse reactions, and precautions to enhance parental medication adherence (26).

(2) Systematic training, education, and capacity building

The training program will adopt a multi-tiered, rolling design. During the initial phase of the intervention, clinical pharmacists will organize three specialized training sessions, each lasting 45 min. The training will cover core knowledge including “Pathogenic Characteristics and Diagnosis of Infectious Diseases in Children” and “Antimicrobial PK/PD Theory and Pediatric Dose Calculation.” A closed-book assessment will follow each session, with a passing score set at 80 points or above to ensure all pediatricians achieve certification. Second, integrate teaching into daily rounds by conducting “micro-training” sessions on specific clinical cases, providing on-site explanations of inappropriate medication practices and corrective measures. Finally, the clinical pharmacist compiles one issue of the Pediatric Rational Medication Newsletter each month. This publication includes case analyses, guideline updates, and relevant data feedback. By distributing it internally within the department and sharing it via WeChat groups, the newsletter continuously reinforces learning outcomes and fosters a departmental culture of rational medication use (27).

(3) Prescription review and process reengineering

Leveraging the hospital’s electronic prescription pre-authorization functionality, establish a dedicated pediatric antimicrobial drug rule repository encompassing multiple rules such as pathogen matching, dosage conversion, standardized treatment duration, and drug interaction contraindications. Embed management requirements into the information workflow to automatically intercept and pop up warning windows for broad-spectrum drugs without diagnostic support, excessive dosages, and inappropriate drug combinations. These alerts prompt physicians to reassess medication regimens or consult with clinical pharmacists before prescribing. Pharmacists at the pharmacy register intercepted prescriptions and provide daily feedback on intercepted cases to clinical pharmacists, thereby establishing a closed-loop prescription review system.

(4) Establish a multidisciplinary collaboration and real-time feedback mechanism

Clinical pharmacists will host a monthly pediatric antimicrobial stewardship meeting, inviting all pediatric physicians to participate. During the meeting, we will address recent instances of inappropriate medication use within the department, share representative case studies, discuss treatment plans for complex cases, and resolve any medication-related questions that may arise for physicians. Additionally, clinical pharmacists established a WeChat work group comprising all pediatricians, pharmacists, and head nurses for non-emergency medication consultations. During working hours, clinical pharmacists provide immediate responses to inquiries and conduct dynamic monitoring, thereby enhancing medication safety and collaborative efficiency.

(5) Strengthen oversight and implement reward and punishment measures

Incorporate pediatric antimicrobial stewardship indicators into departmental and individual performance evaluation systems. Evaluation metrics include antimicrobial utilization rates, usage intensity, rationality rates, and outpatient infusion rates. Infection control nurses conduct monthly inspections of antimicrobial drug usage within the department, while clinical pharmacists analyze and publicly disclose the results of these inspections each month. Physicians demonstrating excellence in rational drug use shall receive performance-based rewards, while those with high frequencies of irrational prescribing practices shall undergo one-on-one counseling and guidance. In severe cases, individual performance bonuses shall be deducted. This incentive-based approach aims to promote standardized medication practices among clinical physicians.

#### 2.2.3 Check

Research and establish a regular monitoring and evaluation mechanism to conduct monthly and quarterly dynamic monitoring of intervention outcomes. Publish statistical results to provide data support for core decision-making. Clinical pharmacists extract core metrics monthly through the HIS system. A monthly Pediatric Antimicrobial Use Quality Analysis Report is generated and distributed to pediatric nurses and physicians, clearly identifying any inappropriate practices observed for each clinician. At the same time, review meetings are held quarterly to analyze issues present in the current phase and clarify improvement directions for subsequent processing stages.

#### 2.2.4 Act

Based on the data analysis conducted during the Check phase, identify the issues that still exist in the data and processes. Research findings indicate that the primary reasons include: (1) Empirical prescribing strategies for certain critical illnesses remain suboptimal. (2) Clinical pharmacist interventions are insufficient during peak outpatient hours and night shifts. (3) Healthcare providers’ awareness and adherence to restricted-use antimicrobial agents have not been fully transformed.

Based on the aforementioned issues, a second round of the PDCA cycle was implemented. While maintaining all interventions from the first round, gaps were identified and addressed to further optimize antimicrobial use among pediatric patients. Key measures include: (1) Strengthen tiered authorization and information-based management of antimicrobial drugs. Prescription authority for “special-use-level” antimicrobial drugs shall be restricted to physicians at the level of associate chief physician or above. For all “restricted-use-level” drugs, a mandatory “prescription application form” will pop up during the prescribing process. Physicians must complete the suspected diagnosis, rationale for use, and planned treatment duration before prescribing. Clinical pharmacists will review and provide feedback on each application in the backend. (2) Establish a WeChat official account group chat to enable efficient and prompt responses during peak outpatient hours and night shifts. (3) Conduct targeted training and feedback based on case studies. Each month, collect 2–3 typical cases of inappropriate medication use. After anonymizing the cases, discuss and analyze them during departmental professional learning sessions. Led by clinical pharmacists, conduct pharmaceutical reviews focused on correcting decision-making biases.

Following the second intervention, the most effective measures demonstrated across both cycles were integrated and institutionalized. Establish a standardized pediatric antimicrobial stewardship system encompassing institutional policies, operational procedures, and organizational structures to lay the foundation for ongoing quality improvement.

### 2.3 Observation indicators

This study aims to provide a clear, evidence-based framework for antimicrobial stewardship (AMS) in pediatrics that is both scalable and sustainable in resource-limited primary hospital settings. To evaluate the effectiveness of this program, the study established primary and secondary outcomes for assessment. All data were extracted directly from the hospital information system without any manual estimates. The specific observation indicators and calculation formulas are as follows:

(1) Antimicrobial usage rate(AUR):

$$\frac{\begin{array}{l} \text{Number of outpatient or inpatient pediatric}\;\\ \text{visits involving antimicrobial}\;\mathrm{use} \end{array}}{\begin{array}{l} \text{Total number of outpatient or inpatient}\;\\ \text{pediatric visits during the same period} \end{array}}\times 100\%$$

(2) Antimicrobial Use in Children: Also known as the Child Defined Daily Dose (cDDD), the Defined Daily Dose (DDD) serves as the unit of measurement. Since the DDD is based on the average daily maintenance dose of the drug for its primary indications in adults, the CDDD values in this study were assigned using a weight-adjusted method. These DDD values were calculated by multiplying the median body weight for the corresponding age group (based on WHO standardized growth curves) by the pediatric daily dose per unit of body weight (g/kg/day) recommended in authoritative guidelines (such as the New Compendium of Chinese Pharmacology and drug labels) for the most common infectious disease indications (28, 29). This calculation method has been used to monitor the intensity of antimicrobial use among children in China. This study divided pediatric patients into four standard age groups, with the median weight for each group based on the World Health Organization Child Growth Standards and data from the National Growth Survey of Chinese Children Aged 0–14: (1) 0–<1 year (9.0 kg); (2) 1–<3 years (13.0 kg); (3) 3–<6 years (18.0 kg); (4) 6–14 years (30.0 kg). Age-specific cDDD values (in g/day) were calculated using the following formula:

$$\begin{array}{c} \text{cDDD}=\text{Unit body weight daily dose}\;(g/\mathrm{kg}/\mathrm{day})\\ \times \text{median body weight for the}\;\mathrm{age}\;\text{group}\;(\mathrm{kg}) \end{array}$$

Complete age-stratified cDDD values for major pediatric antimicrobial agents, stratified by drug and route of administration, are presented in Supplementary Table S3.

(3) Antimicrobial Use Intensity (AUD, Unit: DDDs per 100 person-days): Age-specific cDDD values for pediatric antimicrobial agents have been preconfigured and standardized in the Hospital Information System (HIS) as routine monitoring indicators for pediatric antimicrobial management. For each hospitalized patient, the system automatically identifies the patient’s age at admission and matches it with the corresponding age-specific cDDD value. The HIS system automatically sums the cDDD values for all pediatric inpatients and all antimicrobial agents to calculate the total antimicrobial consumption (in DDDs). The total number of hospital days for pediatric inpatients during the same period is also directly calculated and extracted from the system. The overall antimicrobial usage (AUD) is calculated using the following formula:

$$\frac{\text{Total Antimicrobial consumption}}{\text{Total patient days for pediatric patients during the same period}}\times 100\%$$

All AUD values reported in this study were exported directly from the HIS system. The preset cDDD standard remained consistent throughout the study period, ensuring uniform calculation methods across the baseline period and the two intervention phases, and eliminating bias from manual calculations.

(4) Per capita antimicrobial drug expenditure:

$$\frac{\text{Total Cost of Antibiotics During the Same Period}}{\text{Number of pediatric patients receiving antimicrobial therapy}}$$

(5) Proportion of antimicrobial drug costs:

$$\frac{\begin{array}{l} \text{Total cost of antimicrobial drugs during}\;\text{the}\;\\ {\text{patient}}^{'}s\;\text{hospitalization or outpatient visit} \end{array}}{\begin{array}{l} \text{Total medication costs for inpatient}\;\\ \text{or outpatient care} \end{array}}\times 100\%$$

(6) Antimicrobial outpatient infusion rate:

$$\frac{\begin{array}{l} \text{Number of pediatric outpatient visits receiving}\;\\ \text{antimicrobial drug injections} \end{array}}{\begin{array}{l} \text{Number of pediatric outpatient visits involving}\;\\ \text{antimicrobial}\;\mathrm{use} \end{array}}\times 100\%$$

(7) Rate of irrational prescriptions:

$$\frac{\text{Number of irrational prescriptions}}{\text{Total number of prescriptions containing antimicrobial agents}}\times 100\%$$

Irrational prescriptions are identified through reviews of prescriptions containing antimicrobial agents conducted by clinical pharmacists. These reviews assess various aspects, including compliance with guidelines, appropriateness of indications, drug selection, dosage, route of administration, and duration of therapy.

### 2.4 Statistical analysis

Data analysis was performed using Prism 8.0.1 statistical software. The normality of continuous variables (including per-capita medication costs, number of medications per visit, length of hospital stay, and intensity of antimicrobial use) was assessed using the Shapiro–Wilk test. Although cost-related variables and count variables (such as the number of medications per visit) exhibited moderate right-skewed distributions, given the large sample sizes in both the outpatient and inpatient cohorts (all n > 1,000), the sampling distribution of the sample means approximated a normal distribution. Therefore, independent-samples t-tests were used for between-group comparisons of continuous outcome measures.

Data are presented as mean ± standard deviation (SD), supplemented by the median and interquartile range (IQR) to describe the distribution characteristics of the data. For all proportion-based outcome measures (including antimicrobial usage rates, outpatient antimicrobial infusion rates, inappropriate prescribing rates, and the proportion of antimicrobial costs), data are presented as n (numerator)/N (denominator) (%) with the raw numerator and denominator values explicitly reported. Intergroup comparisons of proportion data were performed using the chi-square test (*χ*^2^ test). For all primary and secondary outcome measures, absolute differences between groups, relative reduction rates, and corresponding 95% confidence intervals (CI) were calculated, and the magnitude of the intervention effect was quantified in conjunction with *p*-values. A two-sided *p*-value < 0.05 was considered statistically significant.

For interrupted time series (ITS) analysis, this paper constructs a piecewise linear regression model to estimate the levels and rates of change in monthly antimicrobial usage during the three study phases (baseline, first PDCA cycle, and second PDCA cycle). The model specification is as follows:

$$\begin{array}{c} Y_{t}=\beta_{0}+\beta_{1}\times {\text{time}}_{t}+\beta_{2}\times \mathrm{int}1_{t}+\beta_{3}\times \text{time}\_\text{after}\_\mathrm{int}1_{t}\\ +\beta_{4}\times \mathrm{int}2_{t}+\beta_{5}\times \text{time}\_\text{after}\_\mathrm{int}2_{t}+\varepsilon_{t} \end{array}$$

*Y_t_ =* antimicrobial usage rate at month *t. time_t_ =* continuous time variable coded in months from the start of the study period, capturing the underlying secular trend. *int1_t_ =* binary indicator for the first intervention point, coded 0 before the first PDCA cycle and 1 from the start of the first PDCA cycle onward, its coefficient *β_2_* represents the immediate level change in the outcome after the first intervention.

t*ime_after_int1t =* continuous time counter for months elapsed since the start of the first PDCA cycle, coded 0 before the first intervention, its coefficient *β_3_* represents the change in slope (trend) after the first intervention relative to baseline.

*int2_t_ =* binary indicator for the second intervention point, coded 0 before the second PDCA cycle and 1 from the start of the second PDCA cycle onward, its coefficient *β_4_* represents the additional immediate level change after the second intervention.

t*ime_after_int2t =* continuous time counter for months elapsed since the start of the second PDCA cycle, coded 0 before the second intervention, its coefficient *β_5_* represents the additional change in slope after the second intervention.

$\epsilon_{t}$ = error term.

The one-month buffer period between the two PDCA cycles (August 2024 and April 2025) was defined as a washout period and excluded from the ITS model. This avoided misclassification of transition-phase data and prevented cross-contamination of effect estimates between the two intervention rounds.

All models were fitted using ordinary least squares regression. Coefficients β2 and β4 were interpreted as the immediate level shifts attributable to each intervention round, while β3 and β5 represented the post-intervention changes in monthly trend relative to the baseline slope.

## 3 Results

### 3.1 Root cause analysis findings

**Figure 1:**
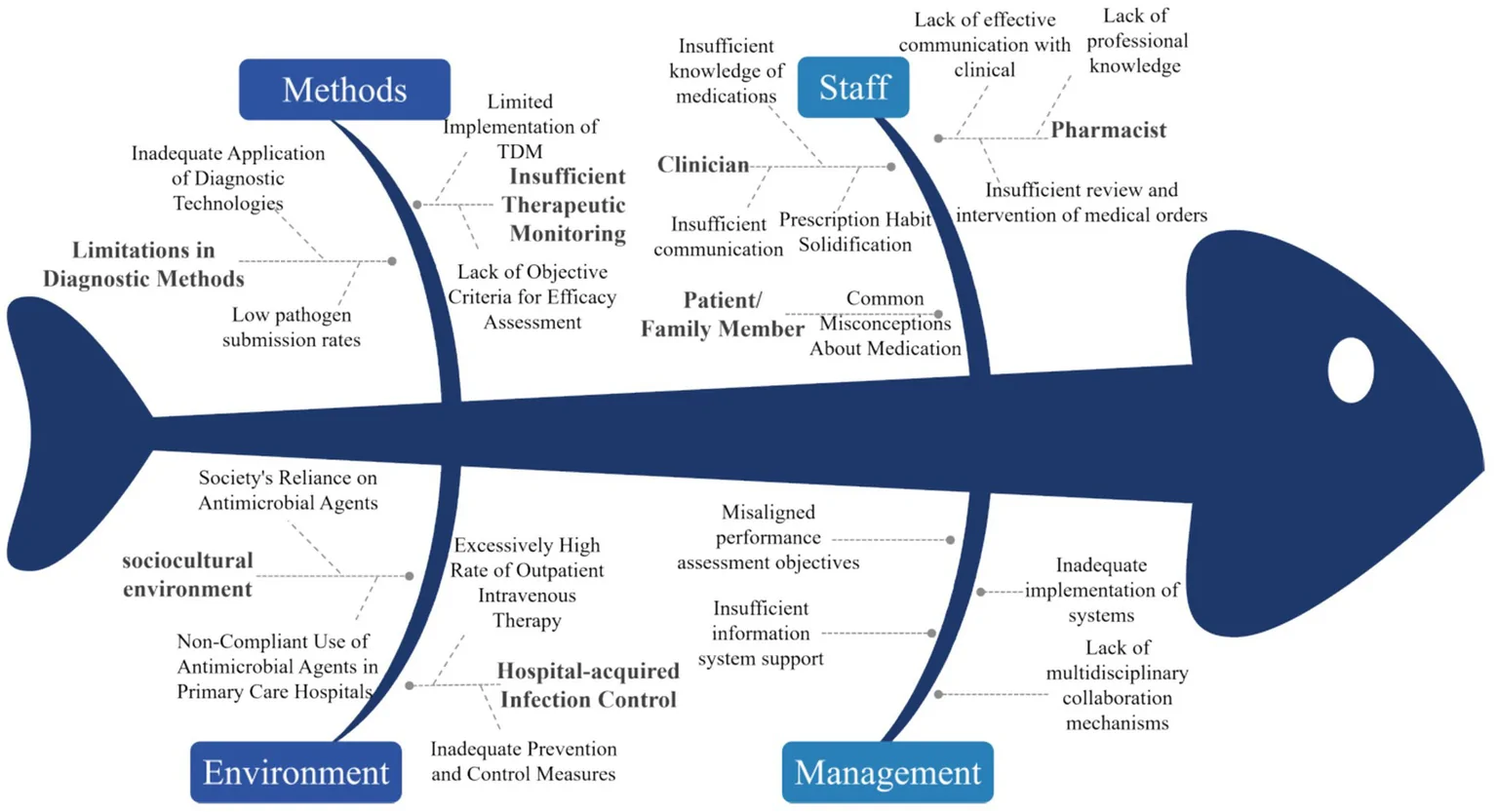
Fishbone diagram: analysis of factors contributing to issues in paediatric antimicrobial use.

To support the root cause analysis, ChatGPT 4.0 was first used to generate a preliminary list of potential factors contributing to the inappropriate use of antimicrobial drugs in children, which served as the basis for drafting the fishbone diagram (Figure 1). Subsequently, a project team composed of pediatricians and clinical pharmacists evaluated all candidate factors against actual clinical practice using a structured questionnaire and scoring process. Ultimately, the clinical team reached a consensus and identified six root causes (Supplementary Table S2). These causes included: inadequate differentiation by physicians between viral and bacterial infections, high rates of empirical antimicrobial use, lax adherence to indications for broad-spectrum antimicrobial drugs and combination therapy, insufficient knowledge among pharmacists, a lack of standardized medication guidelines, an inadequate prescription management system, and insufficient prescription review processes.

Root cause analysis indicates that the core issues underlying the inappropriate use of antimicrobial drugs in pediatric departments of primary hospitals center on two key areas: professional competence of personnel and the establishment of management systems. On the one hand, factors such as limited consultation time at primary hospitals, inadequate diagnostic capabilities for pediatric infectious diseases, and insufficient mastery of antimicrobial stewardship protocols have led to empirical and prophylactic prescribing becoming common clinical practices. On the other hand, primary hospitals lack pediatric-specific antimicrobial stewardship protocols, review systems, and effective intervention mechanisms. Moreover, pharmaceutical services have long been confined to the supply stage, failing to effectively fulfill their core responsibilities of medication review and clinical monitoring. The combined effect of these multiple factors has led to the prominent issue of inappropriate antimicrobial drugs use in pediatrics.

### 3.2 Optimization of antimicrobial use in outpatient settings

**Figure 2:**
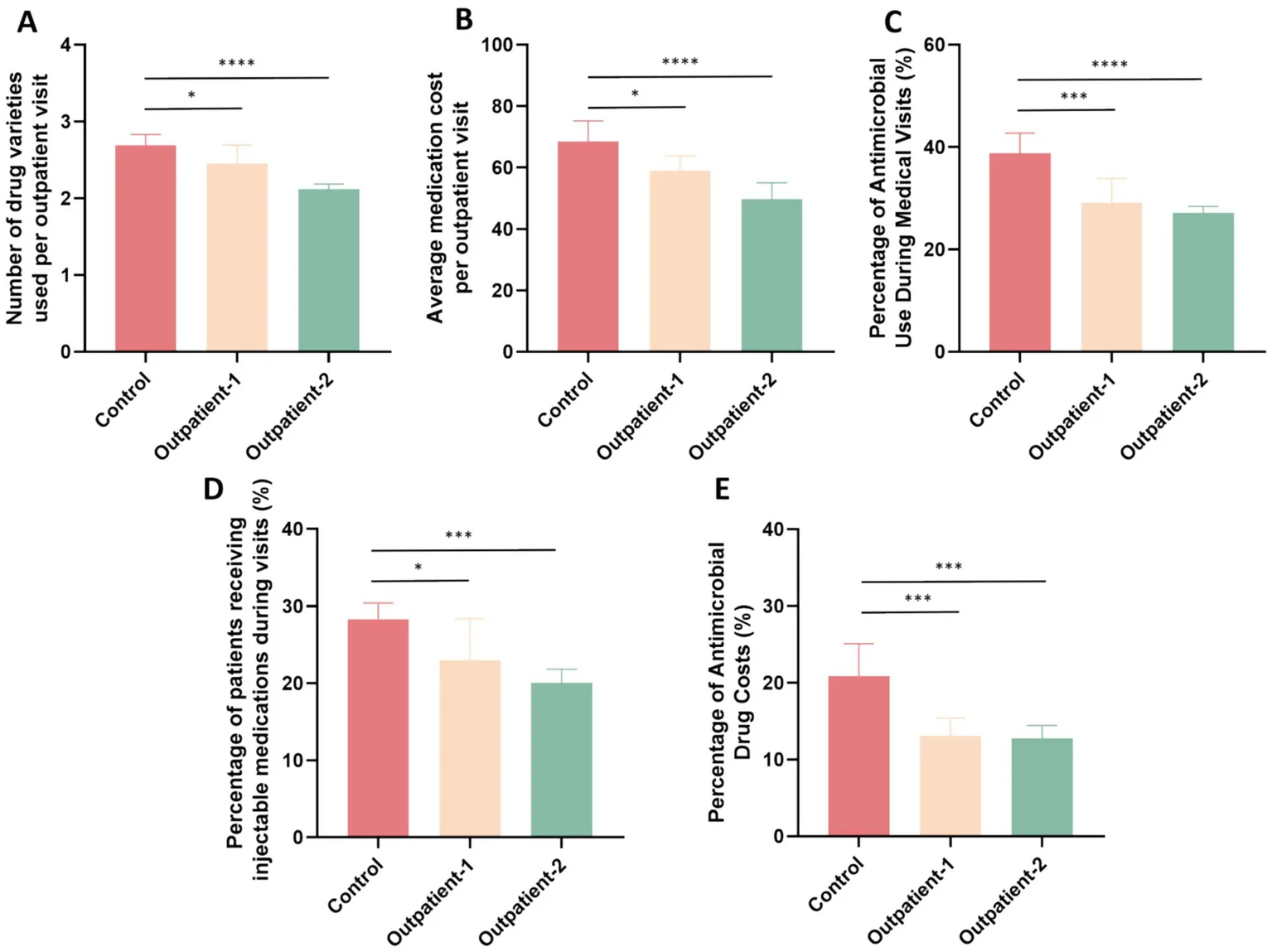
Comparison of indicators between the control group and pediatric outpatient department after two rounds of PDCA cycles. **(A)** Number of drug varieties per outpatient visit. **(B)** Average medication cost per outpatient visit (RMB) (%). **(C)** Percentage of visits with antimicrobial use (%). **(D)** Percentage of antimicrobial injections per visit (%). **(E)** Percentage of antimicrobial drug costs (%) (*p < 0.05, **p < 0.01, ****p < 0.0001 compared with the control group).

The outpatient AUR in the Control was 38.81% (14,416/37145). After one round of PDCA cycle optimization, it decreased to 29.07% (9,639/33158), the absolute differences was −9.74% (95% CI: −10.42 to −9.06), and the relative reduction was 25.10% (*p* < 0.05). Following a second round of optimization, it further decreased to 27.14% (8,663/31917), with an absolute differences of −11.67% (95% CI: −12.33 to −11.01) and a relative reduction of 30.07% (*p* < 0.0001) (Figure 2C). The rate of outpatient antimicrobial drugs infusion decreased from 28.29% (4,078/14416) in the Control to 22.97% (2,214/9639) after the first cycle (absolute difference: −5.32%, relative reduction: 18.81, 95% CI: −6.31 to −4.33, *p* < 0.05) and 20.04% (1736/8663) after the second cycle (absolute difference: −8.25%, relative reduction: 29.16, 95% CI: −9.20 to −7.30, *p* < 0.0001), respectively. This reduction mitigated the potential risks associated with injectable administration and aligned with the rational medication principle of prioritizing oral administration in pediatric outpatient settings (Figure 2D).

Significant optimization was observed in medication cost-related indicators (Figure 2B). The average medication cost per outpatient visit in the Control decreased from ¥68.50 (median: ¥63.41, IQR: 60.71–70.18) before intervention to ¥58.88 (median: ¥57.17, IQR: 54.10–66.29) (absolute difference: -¥9.62, relative reduction: 14.04, 95% CI: -¥10.13 to -¥9.11, *p* < 0.0001) and further to ¥49.73 (median: ¥48.55, IQR: 46.49–52.61) (absolute difference: -¥18.77, relative reduction: 27.40, 95% CI: -¥19.25 to -¥18.29, *p* < 0.0001). The Number of medications used per outpatient visit (Figure 2A) also decreased from 2.68 ± 0.13 (median: 2.73, IQR: 2.58–2.78) in the Control to 2.45 ± 0.21 (median: 2.42, IQR: 2.18–2.71) (absolute difference: -0.23, 95% CI: −0.26 to −0.20, relative reduction: 8.58%, *p* < 0.001) and 2.12 ± 0.24 (median: 2.12, IQR: 2.08–2.19) (absolute difference: -0.56, 95% CI: −0.59 to −0.53, relative reduction: 20.90%, *p* < 0.0001) after two rounds of intervention. The proportion of antimicrobial drug costs decreased from 20.87 to 13.09% (absolute difference: −7.78%, relative reduction: 37.28, 95% CI: −8.12% to −7.44%, p < 0.001) and 12.74% (absolute difference: −8.13%, relative reduction: 38.96, 95% CI: −8.46% to −7.80%, *p* < 0.0001), indicating that the outpatient medication cost structure was significantly optimized after the intervention, effectively reducing patients’ financial burden for medication (Figure 2E).

### 3.3 Optimization of antimicrobial use in inpatient

**Figure 3:**
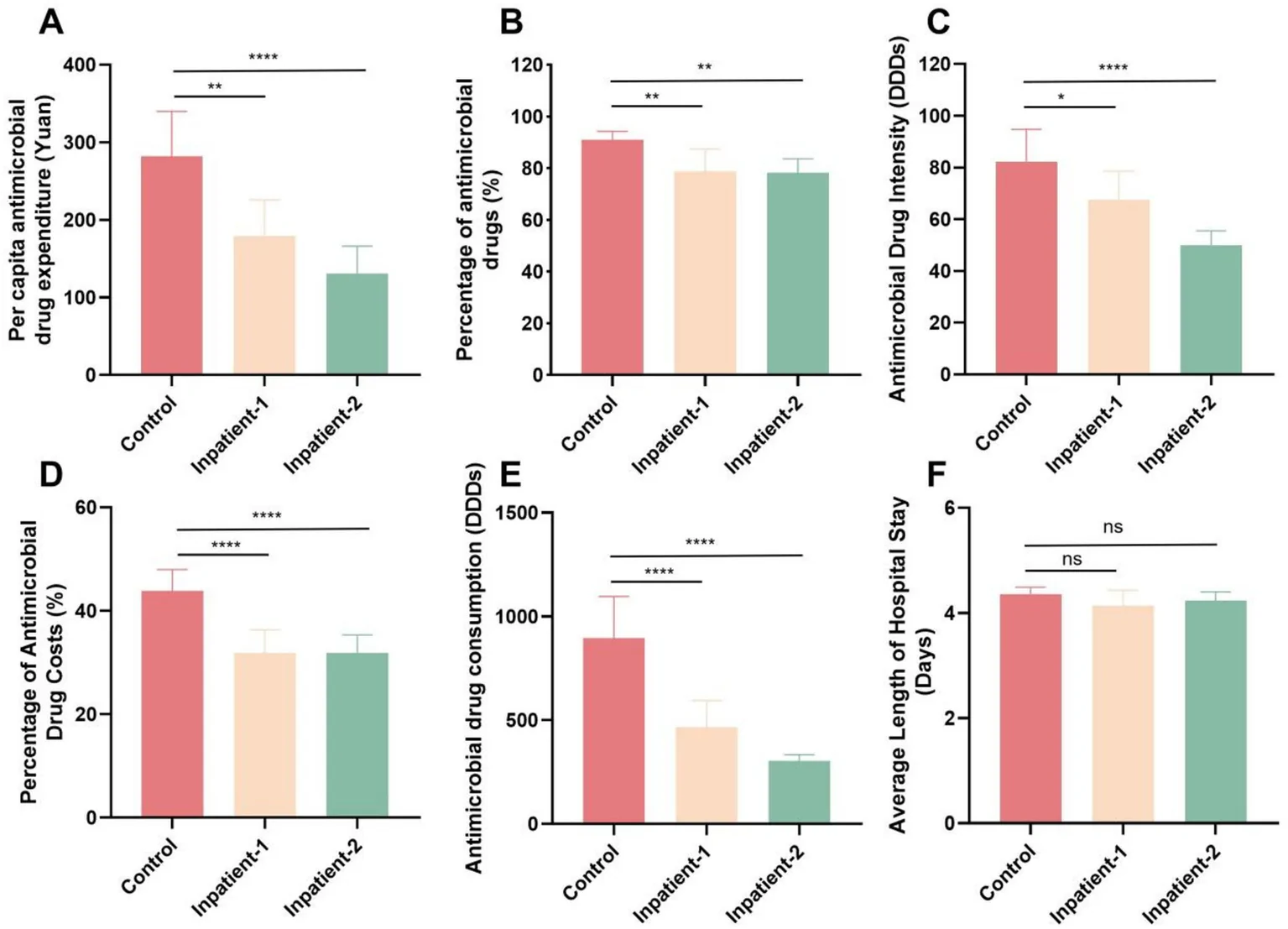
Comparison of indicators between the control group and the pediatric inpatient department after two rounds of PDCA cycles. **(A)** Per capita antimicrobial drugs expenditure (RMB); **(B)** antimicrobial drugs usage rate (%); (C) antimicrobial drugs usage intensity; **(D)** antimicrobial drugs expenditure as a percentage of total drug costs; **(E)** Antimicrobial drugs consumption volume; **(F)** average length of hospital stay (compared to control, *p < 0.05, **p < 0.01, ****p < 0.0001).

As shown in Figure 3A, regarding patient out-of-pocket expenses, the per capita antimicrobial drug cost decreased from ¥281.94 before intervention to ¥179.3 (*p* < 0.01) and ¥130 (*p* < 0.0001) after two rounds of PDCA cycles.

As shown in Figure 3A, regarding patient out-of-pocket expenses, the per capita antimicrobial drug cost was ¥281.94 (median: ¥281.95, IQR: 237.57–307.63) in the Control group. It decreased to ¥179.32 (median: ¥154.01, IQR: 142.97–212.64) after the first PDCA cycle (absolute difference: -¥102.62, relative reduction: 36.40, 95% CI: -¥112.38 to -¥92.86, *p* < 0.001) and ¥130.90 (median: ¥119.82, IQR: 113.31–127.12) after the second cycle (absolute difference: -¥151.04, relative reduction: 53.57, 95% CI: -¥159.83 to -¥142.25, *p* < 0.0001), representing an overall reduction of 53.57% compared with the Control group after two optimization rounds. Additionally, Figure 3B shows that the antimicrobial drugs usage rate decreased from 90.92% (1,392/1531) before intervention 78.36% (992/1266) after two rounds of intervention (absolute difference: −12.59%, relative reduction: 13.85, 95% CI: −15.11% to −10.07%, *p* < 0.0001). Figures 3C,E show that the intensity of antimicrobial use decreased from 82.24 DDDs per 100 person-days before the intervention to 67.56 and 49.94 DDDs per 100 person-days after the two rounds of intervention (*p* < 0.0001), and the total consumption dropped significantly from 894.80 DDDs to 303.31 DDDs following the two rounds of optimization. Moreover, Figure 3D shows that the proportion of antimicrobial drugs expenditure as a percentage of total drug costs decreased from 43.86 to 31.86% (absolute difference: −12.00%, relative reduction: 27.36, 95% CI: −13.52% to −10.48%, *p* < 0.0001), reducing the share of antimicrobial drugs in hospital medication expenses. This optimized the structure of hospital drug expenditures and may support improved efficiency of medical resource utilization.

As a balanced measure of patient safety, this study compared the average length of hospital stay across different phases (Figure 3F). The average length of hospital stay before the intervention was 4.36 days (median: 4.35, IQR: 4.25–4.45), it was 4.14 days (median: 4.05, IQR: 3.73–4.45) after the first PDCA cycle and 4.24 days (median: 4.27, IQR: 4.08–4.35) after the second cycle, with no statistically significant increase observed (all *p* > 0.05). These preliminary results suggest that the reduction in antimicrobial use did not increase the length of hospital stay. However, a stable length of hospital stay alone cannot prove the absence of adverse clinical reactions, nor can it demonstrate the safety of the intervention. Since this study lacks other balanced outcome measures, such as readmission rates, adverse event rates, and nosocomial infection rates, it is not possible to draw definitive conclusions regarding clinical safety. This is also one of the areas requiring further in-depth research.

### 3.4 Time-series analysis of trends in antimicrobial use

**Figure 4:**
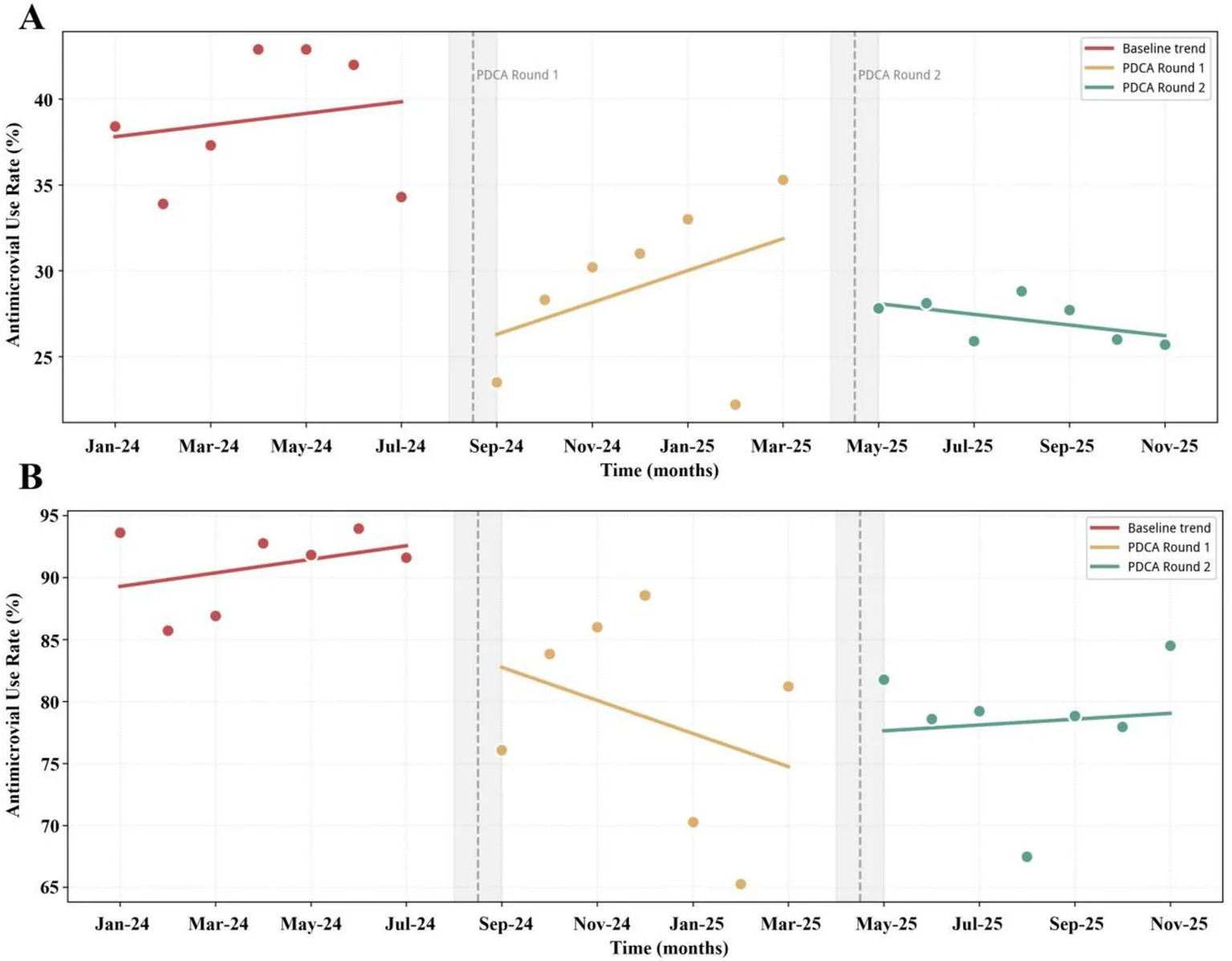
Interrupted time-series analysis of monthly antimicrobial usage rates (AUR) before and after two PDCA cycles. **(A)** Outpatient AUR, **(B)** inpatient AUR. The dashed vertical lines indicate the start of each PDCA cycle. The shaded areas represent the one-month washout periods between phases. The colored lines indicate the results of segmented regression fits for each phase.

To address potential confounding factors associated with seasonal variations in the incidence of respiratory infections, this study conducted a interrupted time series analysis using monthly AUR data covering the entire study period (January 2024 through November 2025). By fitting segmented regression models, we quantified the changes in intercepts and slopes associated with each PDCA cycle (Figure 4).

For outpatients, the baseline AUR was 37.80%, and no significant historical trend was observed (*β* = +0.34%/month, *p* = 0.637). Following the launch of the first PDCA cycle, a significant immediate decrease of 14.81% (95% CI: −24.30 to −5.33, *p* = 0.005) in the AUR level was observed. After the first round of implementation, the trend showed no significant difference compared to baseline (*β* = +0.59% per month, *p* = 0.563). The second PDCA cycle resulted in a further reduction of 4.40%, although this result did not reach statistical significance (*p* = 0.338). Nevertheless, these findings support a temporal association between the implementation of this intervention and a reduction in antimicrobial use, and the overall ITS model was statistically significant (*R*^2^ = 0.736, *p* = 0.0006).

For inpatients, the baseline AUR was 89.27%, and no significant underlying trend was observed (*β* = +0.55% per month, *p* = 0.658). After the first PDCA cycle, there was a 9.01% decline, but this did not reach statistical significance in the ITS model (*p* = 0.257), likely due to the small monthly sample size and high month-to-month variability in the inpatient case mix. The overall model remained statistically significant (*p* = 0.020, *R*^2^ = 0.558).

In summary, analysis of trends over time and in AUR reveals that, both before and during the intervention, AUR was influenced by seasonal respiratory infections, resulting in elevated AUR levels during specific seasons. However, interrupted time series analysis revealed that during the baseline period, no significant downward trend was observed for either inpatient or outpatient AUR. When the PDCA cycle was first initiated, the antibiotic usage rate immediately showed a significant decrease (outpatient AUR: −14.81%, *p* = 0.005). This pattern is more consistent with the effect of the intervention rather than being entirely attributable to seasonal variations.

### 3.5 Analysis of the appropriateness of antibiotic prescriptions

While reducing antibiotic use is certainly an important indicator, appropriate use is equally important. To determine whether the reduction in antimicrobial use was accompanied by an improvement in prescription quality, this study assessed changes in prescription appropriateness across the three study phases. The results are shown in Tables 1, 2.

**Table 1:** Analysis of the rationality of outpatient prescription interventions before and after implementation.

Indicator	Before intervention (*N* = 14,416)	First PDCA cycle (*N* = 9,639)	Second PDCA cycle (*N* = 8,663)
Rate of inappropriate prescriptions	34.75%	21.36%	12.08%
Number of inappropriate prescriptions	5,009	2059	1,047
Off-label use of medications	2,141 (42.74%)	1,153 (56.00%)	596 (56.92%)
The selection of medications is inappropriate	1,232 (24.60%)	354 (17.19%)	197 (18.82%)
Inappropriate dosage and administration	1,212 (24.20%)	378 (18.36%)	194 (18.53%)
Repeated dosing	133 (2.66%)	62 (3.01%)	17 (1.62%)
There are contraindications or adverse interactions	149 (2.97%)	59 (2.87%)	21 (2.01%)
Other medications are not appropriate	142 (2.83%)	53 (2.57%)	22 (2.10%)

Rate of inappropriate prescriptions = Number of prescriptions involving inappropriate use of antimicrobial agents/total number of prescriptions containing antimicrobial agents. For other types of inappropriate prescribing, the first number represents the number of prescriptions for that specific type, and the percentage following it represents the proportion of that type among all inappropriate prescriptions.

**Table 2:** Analysis of the rationale for inpatient prescription interventions before and after implementation.

Indicator	Before intervention (*N* = 1,392)	First PDCA cycle (*N* = 1,024)	Second PDCA cycle (*N* = 992)
Rate of inappropriate prescriptions	40.30%	24.60%	13.42%
Number of inappropriate prescriptions	561	252	133
Off-label use of medications	256 (45.63%)	104 (41.27%)	59 (44.36%)
The selection of medications is inappropriate	108 (19.25%)	48 (19.05%)	26 (19.55%)
Inappropriate dosage and administration	104 (18.54%)	53 (21.03%)	34 (25.56%)
Repeated dosing	29 (5.17%)	17 (6.74%)	4 (3.01%)
There are contraindications or adverse interactions	33 (5.88%)	16 (6.35%)	3 (2.26%)
Other medications are not appropriate	31 (5.53%)	14 (5.56%)	7 (5.26%)

Rate of inappropriate prescriptions = Number of prescriptions involving inappropriate use of antimicrobial agents/total number of prescriptions containing antimicrobial agents. For other types of inappropriate prescribing, the first number represents the number of prescriptions for that specific type, and the percentage following it represents the proportion of that type among all inappropriate prescriptions.

Analysis of outpatient prescription review results showed that the overall rate of inappropriate antimicrobial prescriptions decreased significantly from 34.75% (5,009/14416) at baseline to 21.36% (2059/9639) after the first PDCA cycle (absolute difference: −13.39%, relative reduction: 38.53, 95% CI: −14.32 to −12.46%, *p* < 0.001), and further decreased to 12.08% (1,047/8663) after the second cycle (absolute difference: −22.67%, relative reduction: 65.24, 95% CI: −23.51 to −21.83%, *p* < 0.001). A review of inpatient prescriptions showed that the incidence of inappropriate prescriptions decreased from 40.30% (561/1392) at baseline to 24.60% (252/1024) after the first intervention round (absolute difference: −15.70%, relative reduction: 38.96, 95% CI: −19.01 to −12.39%, *p* < 0.001), and further decreased to 13.42% (133/992) after the second round (absolute difference: −26.88%, relative reduction: 66.70, 95% CI: −29.87 to −23.89%, *p* < 0.001).

An analysis of the number of individual instances of inappropriate prescribing revealed significant decreases across all categories of inappropriate use. However, in both outpatient and inpatient prescriptions, off-label use of antimicrobial agents remained the most common issue, accounting for approximately 50% of all inappropriate prescriptions at every stage. The main reasons for this may include doctors’ overreliance on experience, insufficient updates based on evidence, and lax enforcement of off-label approval and informed consent procedures, among other factors, which indicates that the majority of antimicrobial use in routine clinical practice is unnecessary and without medical indication.

In summary, during all phases of the PDCA implementation, both the total volume of antimicrobial use and the number of inappropriate prescriptions decreased. These data indicate that the decline in antimicrobial use is associated with a reduction in inappropriate prescriptions.

### 3.6 Consolidation of optimization measures

After two rounds of PDCA cycles, this study systematized and standardized intervention measures proven effective through clinical practice. A standardized prescribing process for antimicrobial drugs agents was established for outpatient and inpatient pediatric departments in primary hospitals. The leading role and intervention responsibilities of clinical pharmacists throughout The use of antimicrobial drugs process were clarified, achieving deep integration between pharmaceutical services and clinical care.

**Figure 5:**
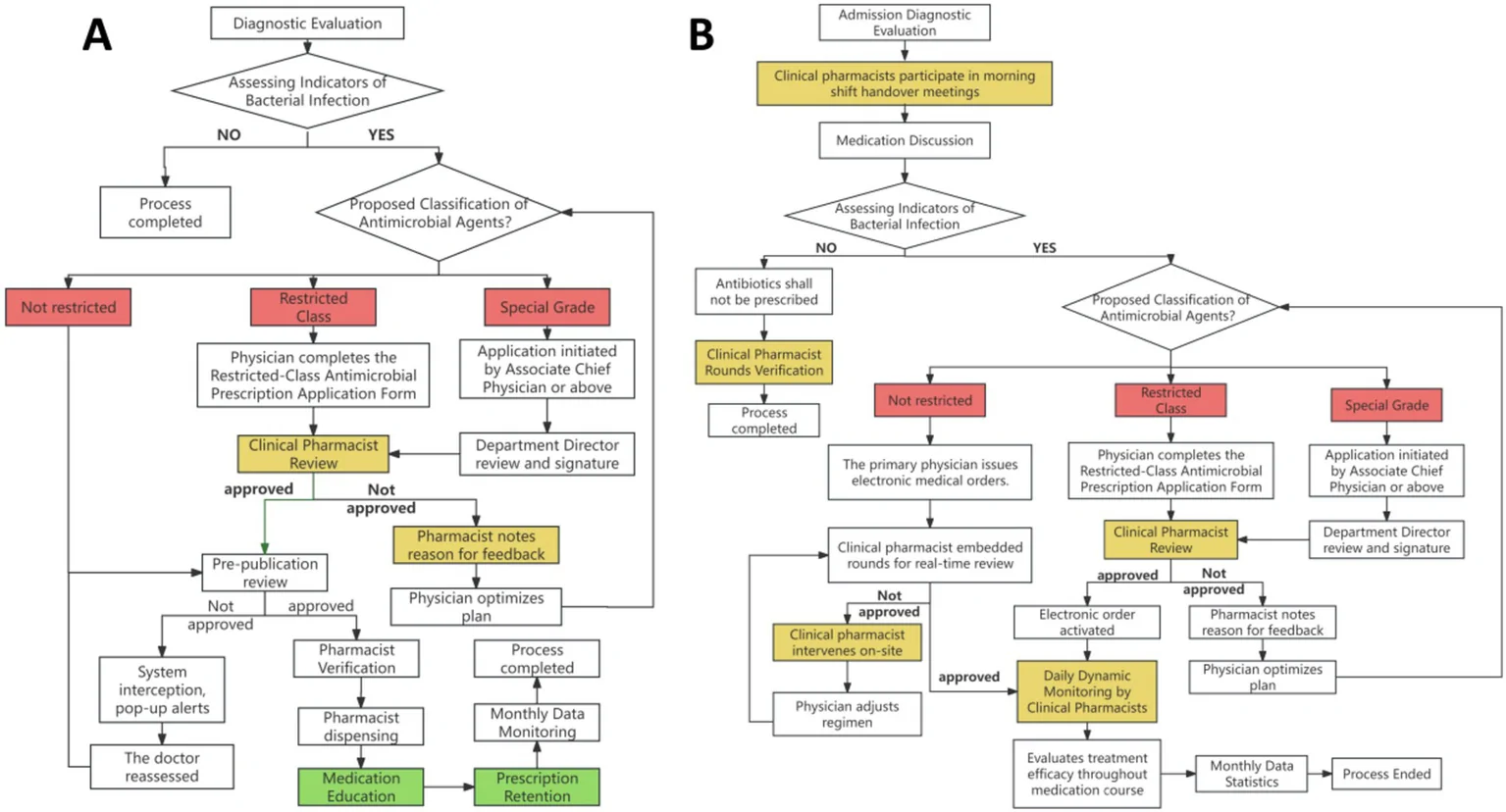
Standardized antimicrobial prescribing process at primary hospitals. **(A)** Outpatient prescribing process. **(B)** Inpatient prescribing process. Yellow indicates steps involving clinical pharmacists. Red indicates the level of antimicrobial drugs prescribed. Green indicates educational outreach steps.

As shown in Figure 5, this process establishes a fully closed-loop management system, defining control requirements for hierarchical authorization, pre-approval review, and end-to-end monitoring. It clearly delineates the full responsibilities of each position. This process features clear operational procedures, well-defined responsibilities, alignment with the characteristics of primary-level hospital care. It offers valuable insights for the scientific and refined management of antimicrobial drugs in pediatric departments at primary hospitals, and provides experience to facilitate the transition from a phased improvement approach to a long-term, standardized antimicrobial management model.

## 4 Discussion

Based on the PDCA cycle theory of quality management, this study developed and implemented a pharmacist-led, AI-assisted antimicrobial stewardship model focused on root cause analysis in the pediatric department of a county-level Grade II Class A general hospital, using antimicrobial usage rates as the primary outcome measure. Following two consecutive PDCA cycle interventions, all observed antimicrobial usage indicators showed sustained and statistically significant improvements. Compared with the first round of intervention, the second round achieved further improvements, consistent with the principles of continuous quality improvement. These results indicate that this management model aligns with the diagnostic characteristics and resource constraints of county-level primary care hospitals, is associated with optimized antimicrobial use, and demonstrates good feasibility and potential effectiveness in managing pediatric antimicrobial use within such healthcare settings. Its implementation logic and core components provide valuable insights for quality improvement in primary care.

The improvement in this study exhibited a distinct stepwise pattern. The findings revealed that the outpatient antimicrobial drugs utilization rate decreased from 38.81 to 29.07% after the first round (*p* < 0.05), and further declined to 27.14% in the second round (*p* < 0.0001). The average antimicrobial drug cost per hospitalized patient decreased from ¥281.94 to ¥130, representing a 53.57% reduction. The antimicrobial AUR dropped from 90.92% before intervention to 78.36% after two rounds of intervention (*p* < 0.0001). AUD decreased from 82.24 before intervention to 67.56 and 49.94 DDDs/per 100 patient-days after two rounds of intervention (*p* < 0.0001). This trend of gradual improvement was associated with the iterative PDCA approach adopted in this study. Through multiple rounds of optimization, gap correction, and integration of measures, significant changes occurred in both clinical prescribing practices and the management system. In particular, the targeted supplementary measures implemented during the second round addressed specific issues—such as insufficient intervention coverage during night shifts and outpatient peak hours, and ineffective enforcement of restricted antimicrobial drug authorizations, which aligns with the core principles of precision quality improvement.

The core issues underlying the misuse of antimicrobial drugs in pediatric departments at primary hospitals primarily revolve around two aspects: insufficient professional competence among staff and an inadequate management system (30). To broaden the scope of root cause analysis, this study introduced ChatGPT 4.0 as an exploratory brainstorming tool to add another dimension to the identification of potential factors. Building on this, a multidisciplinary clinical team combined the “5-3-1” assessment method with actual clinical scenarios to accurately identify the core root causes, thereby enabling subsequent interventions to directly address the key issues. Additionally, through the leadership of clinical pharmacists deeply embedded throughout the entire pediatric diagnosis and treatment process, real-time review of medication indications and personalized medication recommendations are achieved. This study integrates multiple measures, including systematic professional training, review of pediatric-specific electronic prescriptions, and incentive-penalty systems, establishing comprehensive process constraints and institutional safeguards for end-to-end control. Through incremental improvements, we first optimized foundational practices and then addressed gaps in detail. Ultimately, we consolidated and standardized measures proven effective through practice. This transitioned antimicrobial stewardship from a phased upgrade to continuous optimization of pediatric antimicrobial use within the hospital. It is worth noting that this intervention is associated with a reduction in the use of antimicrobial agents and an increase in the rate of appropriate prescribing. The appropriateness rate for outpatient prescriptions rose from 65.25 to 87.92%, while that for inpatient prescriptions increased from 59.70 to 86.58%. The simultaneous optimization of prescription volume and prescription rationality indicates that a pharmacist-led PDCA model can improve the use of antimicrobial agents. However, across all phases, off-label prescribing remained the most common category of inappropriate prescribing, indicating that differential diagnosis and indication verification must continue to be prioritized in future quality improvement cycles. Furthermore, there was no significant increase in length of stay at any stage. Taken together, the improvement in prescription rationality and the stability of length of stay suggest that the decline in antimicrobial use is associated with a reduction in inappropriate prescribing. However, length of stay alone is insufficient as an indicator of clinical safety, in the absence of other patient-level balanced outcome data, it is not possible to draw definitive conclusions regarding the safety of this intervention.

This study was tailored to the characteristics of county-level primary hospitals. It not only fills a gap in research on systematic antimicrobial interventions in primary pediatric diagnosis and treatment but also reinforces the leading role of clinical pharmacists in antimicrobial stewardship at the primary level. It has driven the transformation of pharmaceutical services in primary hospitals from a medication supply model to a clinical service model, thereby fully realizing the professional value of pharmacy in primary diagnosis and treatment.

Nevertheless, this study has certain limitations. First, as a study conducted in a primary hospital, the generalizability of its findings is somewhat limited. Furthermore, the study assessed only short-term improvements in antimicrobial indicators during the study period and did not further analyze the long-term impact of this model on the incidence of nosocomial infections and bacterial resistance in pediatric departments. Second, although we employed interrupted time series (ITS) analysis to control for potential temporal trends, we could not completely rule out the influence of seasonal variations in the incidence of pediatric respiratory infections. Since the control period and the intervention period spanned different calendar months, seasonal fluctuations in disease prevalence would still affect changes in antimicrobial use to some extent. Furthermore, due to limitations in major hospital information systems, which prevented the collection of comprehensive safety indicators such as the rate of unplanned readmissions, the 30-day readmission rate, and the rate of antimicrobial treatment escalation, this study used length of hospital stay as the sole balancing indicator. Although the length of hospital stay remained stable and the appropriateness of prescriptions improved factors that collectively suggest patient safety was not compromised. The lack of these comprehensive balancing indicators indicates that this study cannot completely rule out the possibility of unmeasured adverse outcomes. Additionally, this study applied AI technology only to the root cause analysis phase, due to limited resources, we were unable to deeply integrate it with the hospital’s HIS system to enable intelligent medication review and monitoring. Finally, the scope of the study did not cover more grassroots healthcare institutions, such as township health centers and community health service centers.

In summary, after the management model established in this study was implemented as a targeted, multi-component intervention and institutionalized over the long term in a county-level hospital, the rationality of in-hospital prescribing improved, and the rate of pediatric antimicrobial use, treatment intensity, and patients’ medication burden were all reduced. These findings suggest that the PDCA cycle model established in this study is associated with improved antimicrobial use outcomes, with potential benefits for clinical stewardship practice. The standardised antimicrobial stewardship process has clear operational steps and provides a practical reference framework for county-level hospitals to implement scientific and refined management of paediatric antimicrobial drugs. The clinical pharmacist-led intervention model also offers insights for the transformation of pharmaceutical services in hospitals, with notable clinical reference value. However, as noted above, several limitations remain. Future research could validate the model’s generalisability through multicentre, large-sample studies, extend follow-up periods to assess long-term benefits, and deepen the application of artificial intelligence technology throughout the entire antimicrobial stewardship process. These efforts will further refine the management model and promote its wider implementation in primary healthcare settings.

## 5 Conclusion

This study employed a non-randomized before-and-after controlled study design. Although this design does not allow for definitive causal inferences, it revealed a certain correlation between pharmacist-led PDCA implementation and the optimization of antimicrobial use in pediatrics. In summary, the standardized prescribing process established in this study provides valuable practical insights for optimizing the use of antimicrobial agents in the pediatric departments of county-level general hospitals within the primary health care system. A PDCA cycle project led by clinical pharmacists and supported by artificial intelligence was associated with improved rational drug use in this primary care hospital setting, which suggests potential benefits for similar institutions serving both outpatient and inpatient populations. This approach may also offer reference value for standardizing the management of other high-risk medications in county-level primary care hospitals.

## Data Availability

The original contributions presented in the study are included in the article/Supplementary material, further inquiries can be directed to the corresponding authors.
