# Supplementary material for: The clinical value of PDCA cycle led by clinical pharmacists in optimizing the use of antimicrobial drugs in pediatric departments of primary hospitals
Source: Front Med (Lausanne). 2026 Aug 18;13:1917853. doi: 10.3389/fmed.2026.1917853 (PMC13529994; doi:10.3389/fmed.2026.1917853)
Supplement: Supplementary file 1 [file Table_1.DOCX]

ChatGPT-4.0-Assisted Brainstorming Process and Output Results

1. **Basic Interaction Parameters**

A single, standardized query was submitted to ChatGPT-4.0 via the OpenAI API. The AI underwent no system-level pre-training or fine-tuning, and no follow-up or iterative prompts were provided. The interaction structure is as follows:

Model: ChatGPT-4.0

Date Accessed: August 5, 2024

Number of Interaction Rounds: 1 (single query, single response)

2. **Interaction Structure**

The entire AI-assisted process follows a four-step closed-loop structure, in which there is a clear distinction between AI outputs and human decision-making.

(1) All model and environmental parameters are fixed prior to interaction and remain unchanged throughout the process.

(2) Preset: Predefine prompts, specify the AI’s specialized role, and establish output boundary rules.

(3) Single-query input: Submit a standardized user query containing the complete research background and task requirements.

(4) Raw output generation: Retrieve the complete, unmodified list of candidate factors generated by GPT-4.0.

3. **Complete and Precise Prompts**

（1）Predefined terms:

You are a senior expert in the fields of pediatric antimicrobial stewardship and clinical pharmacy, with 15 years of practical experience in primary healthcare institutions in China. You are thoroughly familiar with the resource constraints, staffing characteristics, clinical practice models, and regulatory requirements of Grade 2A county-level general hospitals. All recommendations must be based on evidence-based medicine, be applicable to real-world pediatric primary care settings, and avoid proposals that are only applicable to tertiary hospitals and would be impractical in primary care settings.

（2）Input for a single query:

**Background:** At a Level 2A general hospital in a county in China, the rate of antimicrobial use in the pediatric outpatient department was 38.81%, while the rate of antimicrobial use among inpatients was 90.92%. The inappropriate and irrational use of antimicrobials is particularly prominent in both outpatient and inpatient settings.

**Task:** Comprehensively identify all potential factors contributing to the irrational use of antimicrobial agents in primary pediatric care. Strictly categorize all factors into the following four dimensions: (1) Personnel factors (including physicians, pharmacists, nurses, patients, and family members); (2) Drug factors (including the characteristics of the drugs themselves, dosage forms, availability, etc.); (3) Environmental factors (including sociocultural factors, policy environment, and healthcare resources); (4) Management/methodological factors (including institutional processes, information systems, and training and evaluation). For each dimension, list no fewer than 10 specific potential factors relevant to the context. Each factor should be a concise noun phrase describing a clear and actionable issue, without redundant explanations.

Output format:

1. Staff

- [Factor 1]

- [Factor 2]

...

2. Medications

- [Factor 1]

- [Factor 2]

...

3. Environment

- [Factor 1]

- [Factor 2]

...

4. Methods

- [Factor 1]

- [Factor 2]

4. **Generated Raw Output Results**

1. Staff

- Insufficient knowledge of medications among physicians

- Poor communication between physicians and patients

- Common misconceptions about medications among patients and their families

- Inadequate professional knowledge among pharmacists

- Insufficient review and intervention of medical orders by pharmacists

- Rigid prescribing habits among physicians

- Insufficient dedicated antimicrobial stewardship training for frontline pediatricians

- High turnover rate of pediatric resident physicians

- Lack of effective communication between pharmacists and clinical staff

- Insufficient continuing medical education on pediatric infectious disease diagnosis

2. Management

- Misaligned performance assessment objectives

- Lack of multidisciplinary collaboration mechanisms

- Lack of standardized ongoing training system for clinical staff

- Lack of regular audit and feedback mechanisms for prescribing practices

- Incomplete hierarchical authorization system for antimicrobial agents

- Inadequate implementation of management systems

- Insufficient performance incentives and penalties for rational drug use

- Insufficient hospital information system support

- Absence of closed-loop management for irrational prescription rectification

- Lack of systematic patient medication education management protocols

3. Environment

- Society's general over-reliance on antimicrobial agents

- Widespread non-compliant antimicrobial use in grassroots primary care

- Excessively high baseline rate of outpatient intravenous therapy

- Inadequate hospital infection prevention and control measures

- Parental pressure for rapid symptom relief requiring antimicrobials

- High seasonal prevalence of pediatric respiratory infectious diseases

- Regional disparities in antimicrobial prescribing norms

- Lack of local public education on rational antimicrobial use

- Pharmaceutical marketing influence on primary care prescribers

- Limited referral pathways for complex pediatric infectious cases

3. Methods

- Inadequate application of diagnostic technologies

- Low pathogen specimen submission rates

- Limited implementation of therapeutic drug monitoring (TDM)

- Lack of objective criteria for efficacy assessment

- Lack of standardized diagnostic pathways for pediatric respiratory infections

- Absence of unified pediatric antimicrobial prescribing guidelines

- Inadequate pre-prescription indication verification processes

- Lack of standardized antimicrobial de-escalation protocols

- Non-standardized documentation of antimicrobial indications in medical records

- Lack of individualized dose calculation protocols for pediatric patients
