# Supplementary material for: The clinical value of PDCA cycle led by clinical pharmacists in optimizing the use of antimicrobial drugs in pediatric departments of primary hospitals
Source: Front Med (Lausanne). 2026 Aug 18;13:1917853. doi: 10.3389/fmed.2026.1917853 (PMC13529994; doi:10.3389/fmed.2026.1917853)
Supplement: Supplementary file 2 [file Table_2.DOCX]

Table S1 Questionnaire for Root Cause Analysis of Inappropriate Antimicrobial Use in Pediatric

| Category | Potential Contributing Factor | Score (5, 3, 1) |
| --- | --- | --- |
| Staff | Insufficient knowledge of medications among physicians |  |
|  | Poor communication between physicians and patients |  |
|  | Rigid prescribing habits among physicians |  |
|  | Common misconceptions about medications among patients and their families |  |
|  | Lack of effective communication between pharmacists and clinical staff |  |
|  | Inadequate professional knowledge among pharmacists |  |
|  | Insufficient review and intervention of medical orders by pharmacists |  |
| Methods | Inadequate application of diagnostic technologies |  |
|  | Low pathogen submission rates |  |
|  | Limited implementation of TDM |  |
|  | Lack of objective criteria for efficacy assessment |  |
| Environment | Society's reliance on antimicrobial agents |  |
|  | Non-Compliant use of antimicrobial agents in primary care Hospitals |  |
|  | Excessively high rate of outpatient intravenous therapy |  |
|  | Inadequate prevention and control measures |  |
| Management | Misaligned performance assessment objectives |  |
|  | Insufficient information system support |  |
|  | Inadequate implementation of systems |  |
|  | Lack of multidisciplinary collaboration mechanisms |  |

Note: This questionnaire was developed based on potential contributing factors generated by GPT-4.0 during an AI-assisted brainstorming phase and is used for expert scoring to identify and confirm the core root causes of inappropriate antimicrobial prescribing. Scoring criteria: 5 points: Extremely important root cause; 3 points: Relatively important root cause; 1 point: General contributing factor. Abbreviation: TDM = Therapeutic Drug Monitoring.
