# Supplementary material for: The clinical value of PDCA cycle led by clinical pharmacists in optimizing the use of antimicrobial drugs in pediatric departments of primary hospitals
Source: Front Med (Lausanne). 2026 Aug 18;13:1917853. doi: 10.3389/fmed.2026.1917853 (PMC13529994; doi:10.3389/fmed.2026.1917853)
Supplement: Supplementary file 3 [file Table_3.DOCX]

Table S2 Root Cause Analysis “5-3-1” Scoring Sheet

| Casuse | Score | | | | | | |
| --- | --- | --- | --- | --- | --- | --- | --- |
|  | Member1 | Member2 | Member3 | Member4 | Member5 | Member6 | Total |
| Staff | | | | | | | |
| Insufficient knowledge of medications among physicians | 5 | 5 | 5 | 5 | 5 | 5 | 30 |
| Poor communication between physicians and patients | 1 | 1 | 3 | 3 | 1 | 1 | 10 |
| Rigid prescribing habits among physicians | 5 | 5 | 5 | 5 | 5 | 5 | 30 |
| Common misconceptions about medications among patients and their families | 3 | 5 | 5 | 5 | 3 | 5 | 26 |
| Lack of effective communication between pharmacists and clinical staff | 3 | 3 | 3 | 3 | 3 | 3 | 18 |
| Inadequate professional knowledge among pharmacists | 5 | 5 | 3 | 3 | 3 | 3 | 22 |
| Insufficient review and intervention of medical orders by pharmacists | 5 | 5 | 5 | 5 | 5 | 3 | 28 |
| Methods | | | | | | | |
| nadequate application of diagnostic technologies | 3 | 3 | 1 | 3 | 1 | 1 | 12 |
| Low pathogen submission rates | 3 | 3 | 3 | 3 | 3 | 3 | 18 |
| Limited implementation of TDM | 3 | 5 | 3 | 5 | 5 | 5 | 26 |
| Lack of objective criteria for efficacy assessment | 5 | 3 | 1 | 3 | 5 | 1 | 18 |
| Environment | | | | | | | |
| Society's reliance on antimicrobial agents | 3 | 3 | 1 | 3 | 3 | 3 | 16 |
| Non-Compliant use of antimicrobial agents in primary care hospitals | 5 | 5 | 3 | 5 | 3 | 3 | 24 |
| Excessively high rate of outpatient intravenous therapy | 3 | 3 | 5 | 3 | 3 | 3 | 20 |
| Inadequate prevention and control measures | 3 | 3 | 1 | 3 | 1 | 1 | 12 |
| Management | | | | | | | |
| Misaligned performance assessment objectives | 3 | 3 | 3 | 1 | 1 | 5 | 16 |
| Insufficient information system support | 3 | 3 | 5 | 3 | 1 | 1 | 16 |
| Inadequate implementation of systems | 3 | 5 | 5 | 5 | 5 | 3 | 26 |
| Lack of multidisciplinary collaboration mechanisms | 5 | 5 | 5 | 5 | 3 | 5 | 28 |

Members assigned scores of 1 (unimportant), 3 (moderately important), and 5 (very important) to evaluate each factor. A total of six members participated in the “5-3-1” scoring process, with a maximum total score of 30 points. Abbreviation: TDM = Therapeutic Drug Monitoring.
