# Supplementary material for: The clinical value of PDCA cycle led by clinical pharmacists in optimizing the use of antimicrobial drugs in pediatric departments of primary hospitals
Source: Front Med (Lausanne). 2026 Aug 18;13:1917853. doi: 10.3389/fmed.2026.1917853 (PMC13529994; doi:10.3389/fmed.2026.1917853)
Supplement: Supplementary file 4 [file Table_4.DOCX]

Table S3. cDDD Values for Major Antimicrobial Agents by Age Group

| Antimicrobial Agent | Route | Recommended daily dose (g/kg/day, median of routine maintenance dose) | cDDD by Age Group (g/day) | | | |
| --- | --- | --- | --- | --- | --- | --- |
|  |  |  | 0–<1 y  (9.0 kg) | 1–<3 y  (13.0 kg) | 3–<6 y  (18.0 kg) | 6–14 y  (30.0 kg) |
| Amoxicillin | Oral | 0.03 | 0.27 | 0.39 | 0.54 | 0.9 |
| Amoxicillin-clavulanate | Oral | 0.03 (amoxicillin component) | 0.27 | 0.39 | 0.54 | 0.9 |
| Cefuroxime axetil | Oral | 0.025 | 0.225 | 0.325 | 0.45 | 0.75 |
| Cefuroxime sodium | Intravenous | 0.075 | 0.675 | 0.975 | 1.35 | 2.25 |
| Ceftriaxone sodium | Intravenous | 0.05 | 0.45 | 0.65 | 0.9 | 1.5 |
| Ceftazidime sodium | Intravenous | 0.075 | 0.675 | 0.975 | 1.35 | 2.25 |
| Cefmetazole sodium | Intravenous | 0.075 | 0.675 | 0.975 | 1.35 | 2.25 |
| Latamoxef sodium | Intravenous | 0.06 | 0.54 | 0.78 | 1.08 | 1.8 |
| Azithromycin | Oral/Intravenous | 0.01 | 0.09 | 0.13 | 0.18 | 0.3 |
| Clindamycin hydrochloride | Intravenous | 0.025 | 0.225 | 0.325 | 0.45 | 0.75 |

Note: cDDD = pediatric defined daily dose. The selected dose represents the median routine maintenance dose of the drug when used in children, which is consistent with the World Health Organization (WHO) definition of DDD—the average maintenance dose for the primary indication. The cDDD values for the main antimicrobial agents in this study were extracted directly from the hospital information system (HIS).
